# Thermal Tolerance of the Coffee Berry Borer *Hypothenemus hampei*: Predictions of Climate Change Impact on a Tropical Insect Pest

**DOI:** 10.1371/journal.pone.0006487

**Published:** 2009-08-03

**Authors:** Juliana Jaramillo, Adenirin Chabi-Olaye, Charles Kamonjo, Alvaro Jaramillo, Fernando E. Vega, Hans-Michael Poehling, Christian Borgemeister

**Affiliations:** 1 Institute of Plant Diseases and Plant Protection, University of Hannover, Hannover, Germany; 2 International Center of Insect Physiology and Ecology (icipe), Nairobi, Kenya; 3 Centro Nacional de Investigaciones de Café, Manizales, Colombia; 4 Sustainable Perennial Crops Laboratory, United States Department of Agriculture, Agricultural Research Service, Beltsville, Maryland, United States of America; University of Bristol, United Kingdom

## Abstract

Coffee is predicted to be severely affected by climate change. We determined the thermal tolerance of the coffee berry borer , *Hypothenemus hampei*, the most devastating pest of coffee worldwide, and make inferences on the possible effects of climate change using climatic data from Colombia, Kenya, Tanzania, and Ethiopia. For this, the effect of eight temperature regimes (15, 20, 23, 25, 27, 30, 33 and 35°C) on the bionomics of *H. hampei* was studied. Successful egg to adult development occurred between 20–30°C. Using linear regression and a modified Logan model, the lower and upper thresholds for development were estimated at 14.9 and 32°C, respectively. In Kenya and Colombia, the number of pest generations per year was considerably and positively correlated with the warming tolerance. Analysing 32 years of climatic data from Jimma (Ethiopia) revealed that before 1984 it was too cold for *H. hampei* to complete even one generation per year, but thereafter, because of rising temperatures in the area, 1–2 generations per year/coffee season could be completed. Calculated data on warming tolerance and thermal safety margins of *H. hampei* for the three East African locations showed considerably high variability compared to the Colombian site. The model indicates that for every 1°C rise in thermal optimum (T_opt._), the maximum intrinsic rate of increase (*r*_max_) will increase by an average of 8.5%. The effects of climate change on the further range of *H. hampei* distribution and possible adaption strategies are discussed. Abstracts in Spanish and French are provided as supplementary material Abstract S1 and Abstract S2.

## Introduction

The impact of climate change on natural systems has emerged as one of the most critical issues faced by humankind. According to the Intergovernmental Panel on Climate Change (IPCC) [1], an increase in the mean global temperature of 1.4° to 5.8° C is expected by the end of the 21^st^ century [2]. IPCC [1] provides an overview of our scientific understanding on climate change, and this assessment offers evidence of impact on, among others, natural biological systems [1]. Global climate change is likely to directly influence the dynamics of all trophic levels and further disrupt the multitrophic interactions among the different communities [3], [4].

In addition, climate change represents an immediate and unprecedented threat to agriculture. A 10–20% decline in overall global crop yields is predicted by 2050 [1]. This is of particular importance for crops such as coffee, which serves as the economic foundation for many countries in the tropics, and on which millions of people depend for their subsistence. Out of 103 species in the genus *Coffea* (Rubiaceae), only two are commercially traded: *C. arabica* L. and *C. canephora* Pierre ex A. Froehner [5], [6]. In terms of monetary value, coffee is the most heavily traded commodity in the world after oil [7]. Around 70% of the world's coffee is produced by small-scale farmers, with over 20 million coffee-farming families – equivalent to more than 100 million people - depending on its production for their subsistence [8]. Recent studies from Brazil, Mexico and Uganda show that even minimal increases in the mean temperature due to climate change will have disastrous consequences for coffee production, in some cases reducing the area presently suitable for coffee production by up to 95% [9]–[11]. *Coffea canephora* (widely known as robusta coffee) is native to humid forests or the lowland forests of the Congo River Basin, an area with elevations ranging from 0–1,200 meters above sea level (m.a.s.l.) [5], and an average temperature of 24–26°C [12]. *Coffea arabica*, regarded as the highest quality coffee, is native to the highlands of South Western Ethiopia where it grows naturally as an understory tree in forests at elevations ranging from 1,600–2,800 m.a.s.l. [5], and an average temperature of 18–21°C [13]. Above or below these temperatures the yield and quality of *C. arabica* is greatly reduced [14], [15]. Of the total world coffee production, 60% is arabica coffee [16]. Since the vast majority of *C. arabica* and *C. canephora* are grown in the tropics they are especially vulnerable to global climate change [17]. Climate-induced stress may render plants more vulnerable to opportunistic herbivores [18]. Furthermore, the direct effects of temperature on herbivores are likely to be larger and more important than any other factor associated with climate change like drought, CO_2_ levels, etc. [19]–[21]. Effects of climate change on insect herbivores can be direct, through impacts on their life history traits and number of generations per year [22], phenology [23], winter mortality [24] and distribution range [25], or indirect, e.g., when host-parasitoid interactions are affected [26] or when insects respond to climate-induced changes on the host plant [27].

**Figure 1:**
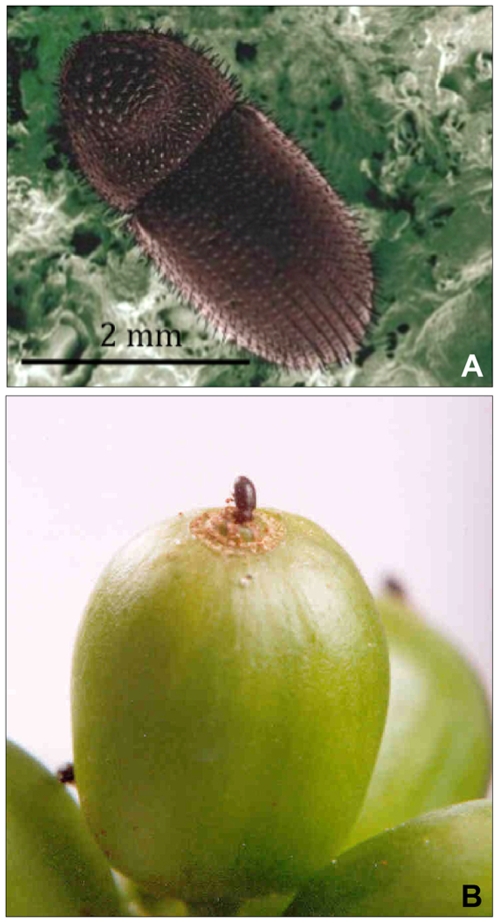
Female of the coffee berry borer (a), and female *Hypothenemus hampei* penetrating a coffee berry (b); (Photos: (a) Eric Erbe (USDA, ARS); (b) Gonzalo Hoyos CENICAFE).

Knowledge on thermal tolerance is essential to predict the effects of climate change in an organism [28]. Such information has never been used to predict the effects of climate change, i.e., global warming on the coffee berry borer *Hypothenemus hampei* Ferrari (Coleoptera: Curculionidae: Scolytinae) the most important pest of coffee throughout the world [29], [30] (Fig. 1). In this paper, we determine the thermal tolerance for *H. hampei* and make some inferences on the effects of climate change on the pest and on coffee production in the tropics, using climatic data from four coffee producing areas in Africa and South America. In addition, the original host plant and the possible area of origin of *H. hampei* as well as the reasons for the absence of the *H. hampei* in the presumed area of origin of *C. arabica* are discussed.

## Results

### Effect of temperature on the colonization of coffee berries and mortality of H. hampei colonizing females

**Figure 2:**
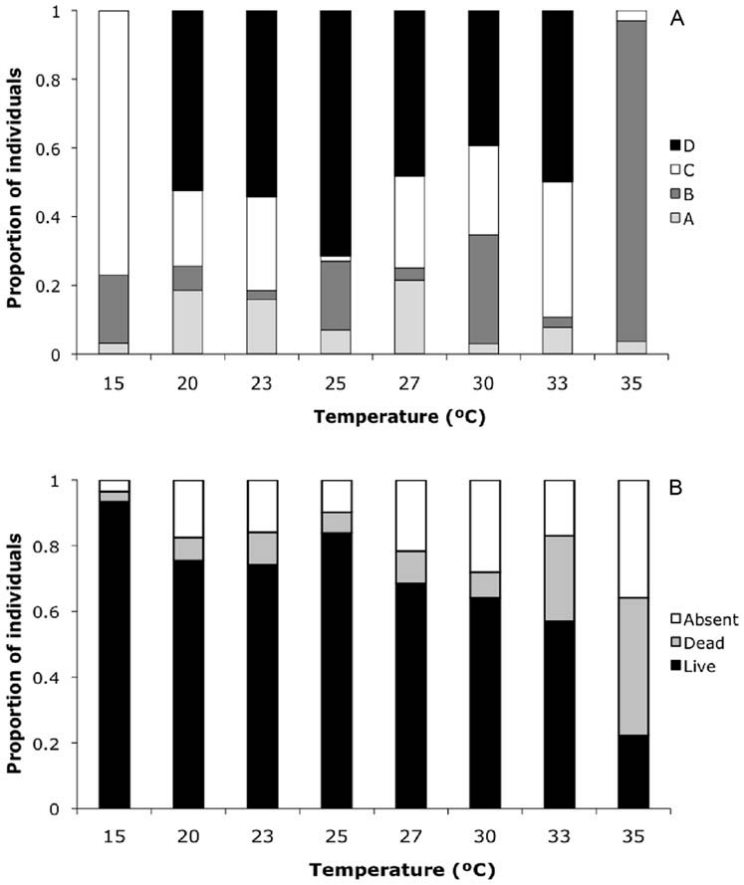
*Hypothenemus hampei* colonizing females, % in positions inside the berry (a); % mortality or failure to penetrate the coffee berry (b).

The proportion of colonizing *H. hampei* females in the different positions inside the berries and their mortality/survival as a function of temperature are presented in Fig. 2. Across temperatures, <25% of colonizing females failed to penetrate the berries (position A) (Fig. 2a). In general the Logistic Model gave a good fit to the data for both the position of the colonizing females inside the berries (χ_14_^2^ = 2662.19, *P*<0.0001) and mortality cases (χ_7_^2^ = 211.87, *P*<0.0001).

The position of the *H. hampei* females in the berries was significantly affected by temperature (χ_7_^2^ = 953.92, (7, N = 5099) *P*<0.0001). The highest proportion of *H. hampei* females found in position D was recorded at 25°C (74.0%), followed by 23°C (54.2%), 20°C (52.3%) and 33°C (49.7%) (Fig. 2). At 15°C and 35°C there was no oviposition and the proportion of females found in the position B and C were 19.8%, 76.9% and 93.3%, 3.0%, respectively, indicating that at 15°C the colonizing females reached the endosperm but did not oviposit. On the other hand at 35°C the colonizing females did not reach the endosperm and remained in position B (Fig. 2a). Likewise, the temperature significantly affected the mortality/survival (χ_7_^2^ = 546.15, (7, N = 5099) *P*<0.0001). The highest numbers of live females were found at 15°C (93.4%), followed by 25°C (83.8%) and 20°C (75.4%) (Fig. 2b). The highest numbers of dead *H. hampei* females were recorded at 35°C followed by 33°C (41.9% and 26.0%, respectively). In general, the proportion of surviving *H. hampei* females was high from 15 to 25°C; at higher temperatures (27 and 30°C), survival started to decrease considerably (Fig. 2b).

### Effect of temperature on the developmental rate of H. hampei

None of the *H. hampei* life stages developed successfully at 15 and 35°C. The youngest life stages (egg and L1) developed between 20–33°C, whereas second instar larvae, prepupa, pupa and adult developed only between 20–30°C (Table 1). For all *H. hampei* life stages, the development time decreased significantly with temperature (between 20–30°C for egg, larvae 1 and 2; and 20–27°C for later life stages). At 33°C females oviposited but subsequent dissections revealed that 95% of the L1 died after eclosion. The developmental time of *H. hampei* immature stages was significantly influenced by temperature (Table 1). The duration of all immature stages except L1 (*F*_4, 15_ = 4.92, *P* = 0.0161), was significantly longer at 20°C than at 23, 25, 27 and 30°C, i.e. for egg (*F*_4, 15_ = 29.51, *P*<0.0001), L2 (*F*_4, 15_ = 39.0, *P*<0.0001), pre-pupa (*F*_4, 15_ = 8.65, *P* = 0.0021), pupa (*F*_4, 15_ = 22.40, *P*<0.0001). Egg to adult developmental time differed significantly at all temperatures tested (*F*_4, 15_ = 305.88, *P*<0.0001) (Table 1).

**Table 1:** Mean (±se) developmental time (in days) of *Hypothenemus hampei* life stages at different temperatures.

Life stages	Temperature (°C)							
	15*	20	23	25	27	30	33**	35*
Eggs	–	12.0±0.6a	7.7±0.3b	5.3±0.3c	4.3±0.3dc	3.3±0.3d	4.7±0.3dc	–
Larva I	–	6.3±1.3a	3.3±0.9b	2.8±0.5b	2.0±0.1b	1.7±0.3b	9.0±0.6c	–
Larva II	–	9.0±0.6a	6.0±0.6b	5.8±1.1b	5.0±0.6b	4.0±0.6b	–	–
Pre-pupa	–	12.7±0.7a	7.7±1.2b	6.0±0.4b	5.0±0.6b	5.3±1.2b	–	–
Pupa	–	16.3±1.4a	6.5±0.3b	6.3±0.5b	5.2±0.3b	6.0±0.7b	–	–
Egg to adult	–	53.7±0.7a	31.2±0.4b	26.6±0.5c	21.8±0.3d	23.3±0.3e	–	–

Within a row, means followed by the same letter are not significantly different (*P* = 0.05), Student-Newman-Keuls test.

* *H. hampei* oviposition was not recorded at these temperatures.

** *H. hampei* oviposition took place at this temperature but the first instar larvae died after eclosion.

For all beetle life stages, significant relationships between the developmental rate and temperatures were recorded (Table 2). In egg, pre-pupa, pupa and egg to adult time, the relationships were strongly linear, whereas a weaker relationship was recorded for L1, and linear regressions did not yield a good fit for development of the L2 (Table 2). For egg to adult, the lower developmental threshold was 14.9°C and the thermal requirement for completion of the pre-reproductive phase was calculated as 262.47 degree-days above the lower developmental threshold (Table 2).

**Table 2:** Estimates of the linear regression analyses (N = 15), lower thermal thresholds and the thermal constants for *Hypothenemus hampei* life stages.

Life stages	Linear range (°C)	Regression Equations a	*r^2^*	*F*	P>F	To^b^	Kc^c^
Eggs	20–30	Y = −0.37713+0.02265*T	0.91	152.91	<0.0001	16.7	44.15
Larva I	20–30	Y = −0.78949+0.04815*T	0.57	21.05	0.0004	16.4	20.77
Larva II*		Y = −0.13038+0.01249*T	0.30	7.39	0.0166	10.4	80.06
Pre pupae	20–27	Y = −0.27788+0.01791*T	0.75	38.08	<0.0001	15.5	55.83
Pupa	20–27	Y = −0.29549+0.01861*T	0.66	50.46	<0.0001	15.9	53.73
Egg-adult	20–27	Y = –0.05689+0.00381*T	0.97	861.15	<0.0001	14.9	262.47

a Calculated after Campbell et al. [53], where X is the temperature (°C) and Yis the developmental rate (1/developmental time).

b Lower development threshold (°C).

c Thermal constant (in day degrees).

* linear regressions did not yield a good fit for development of the L2.

**Figure 3:**
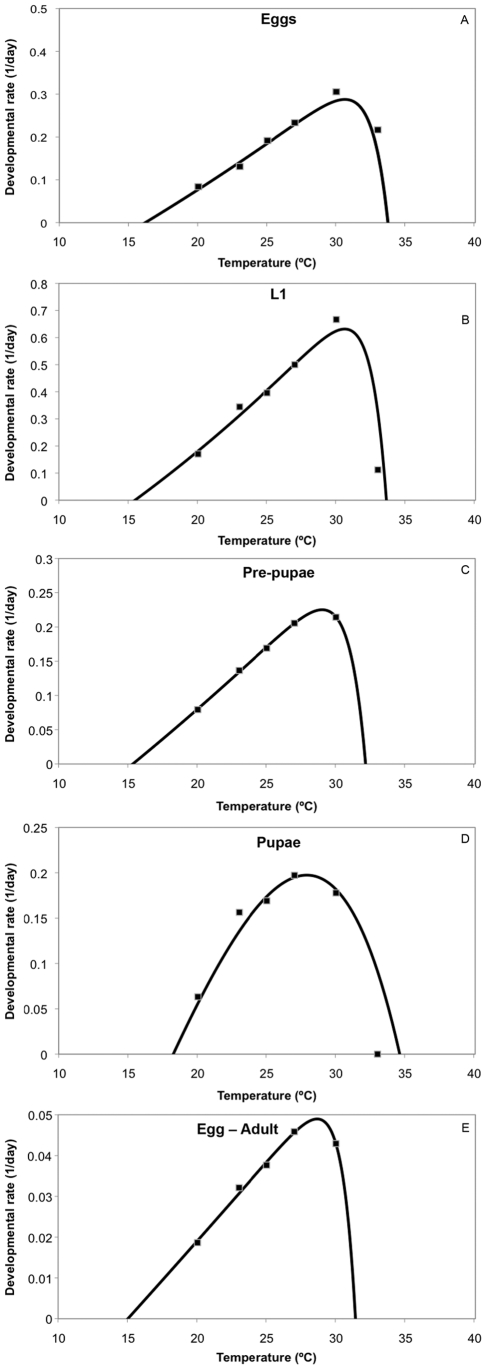
Effect of temperature on the developmental rates of *Hypothenemus hampei.*

Developmental rates increased linearly between 15 and 27°C for prepupa, pupa and adult, and between 15 and 30°C for eggs and L1 (Fig. 3). In general, the non-linear model gave a good fit to the data sets within a range of 20–27°C for L1, pre-pupae, pupae and egg to adult development, and between 20–30°C for eggs and L1 (Fig. 3).

The fitted parameters of the model are presented in Table 3. Based on the non-linear models, the optimum temperature for the development of *H. hampei* egg and L1 was estimated as 30–32°C and for L2, pre-pupa, pupa and adult between 27–30°C. The lower and upper developmental threshold for all life stages was estimated as 14.9 and 32°C, respectively (Table 2 and Fig. 3).

**Table 3:** Fitted parameters of the non-linear modified Logan model [84], [85] for *Hypothenemus hampei* life stages.

Life stages	Parameters						
	ρ	*T_max_*	Δ	λ	*r^2^*	*F*	*P*>*F*
Eggs	0.0153	35.5	1.198	−1.2806	0.90	75.51	<0.0001
Larva I	0.0254	34.9	0.1537	−1.4805	0.70	18.92	<0.0001
Pre pupae	0.0135	34.133	1.23	−1.2296	0.56	5.02	0.0176
Pupa	0.0374	43.7705	10.2205	−1.5557	0.63	15.76	0.0001
Egg-adult	0.00358	34.2548	0.1537	−1.0551	0.97	330.31	<0.0001

### Life table parameters of H. hampei

Life table parameters are presented in Table 4. The intrinsic rate of increase (r_m_) was significantly higher at 25 and 27°C. Similarly, the reproductive rate (R_0_) significantly differed among temperatures tested, and was highest at 25°C followed by 27°C. The lowest reproductive rate was recorded at 30°C. With 68.0 and 11.8 days, the maximum generation time (*G*) and the doubling time (*t*), respectively, were obtained at 20°C. The finite rate of increase (λ) remained almost constant at all temperatures tested (Table 4).

**Table 4:** Average (+ se) population growth parameters of *Hypothenemus hampei* at five constant temperatures.

Parameter	Temperature (°C)				
	20	23	25	27	30
r_m_	0.06±0.002a	0.10±0.007a	0.14±0.008b	0.14±0.0053b	0.10±0.028a
R_0_	54.0±7.4a	67.9±20.2a	146.6±31.8a	84.5±26.38ab	23.1±12.9b
G	68.0±1.2	40.9±0.24	35.5±1.0	32.76±2.82	30.6±0.8
λ	1.06±0.002	1.10±0.01	1.15±0.009	1.14±0.01	1.10±0.031
*t*	11.8±0.24	6.8±0.45	4.9±0.3	5.1±0.2	6.8±2.0

Means followed by the same letter within rows are not significantly different (P = 0.05, Student-Newman-Keuls sequential test). r_m_, intrinsic rate of natural increase; R_0_, net reproductive rate; G, mean generation time (days); λ, finite rate of increase; *t*, doubling time (days).

### Effect of temperature on fecundity of H. hampei

Pre-oviposition period and total *H. hampei* fecundity were significantly affected by temperature (*F*_4, 14_ = 8.08, *P* = 0.0035) and (*F*_4, 14_ = 40.97, *P*<0.0001), respectively. The longest pre-oviposition periods were recorded at 20°C and 23°C. Total fecundity measured as number of eggs laid by colonizing females before oviposition of the F1 females was significantly higher at 20°C (296.9 eggs) and lowest at 30°C (64.3 eggs) (Table 5). No differences were recorded in sex ratio, which ranged from 0.84 to 0.9 for all temperatures tested.

**Table 5:** Mean (±se) of pre-oviposition period, total fecundity, daily fecundity and sex ratio of *Hypothenemus hampei* at constant temperatures.

Parameters	Temperature (°C)				
	20	23	25	27	30
Pre-oviposition period (days)	5.7±0.3a	4.0±0.0ab	3.3±0.3b	3.7±0.3b	3.0±0.6b
Total fecundity*	296.94±9.4a	199.6±13.8b	201.5±19.4b	160.0±11.6b	64.3±8.4c
Sex ratio**	0.9±0.07a	0.85±0.03a	0.9±0.004a	0.84±0.2a	0.9±0.1a

Means followed by the same letter within rows are not significantly different (P = 0.05, Student-Newman-Keuls sequential test).

* Total number of eggs laid per female at a given temperature.

** Calculated as the proportion of *H. hampei* females in the total population.

### Estimated number of *H. hampei* generations, warming tolerance and thermal safety margins in four coffee growing locations in East Africa and South America

**Figure 4:**
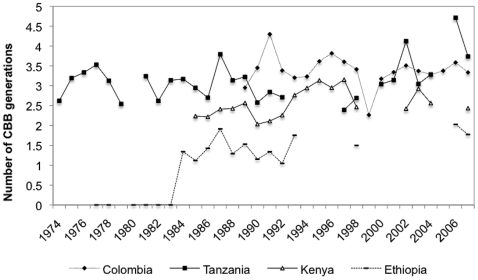
Generations of *Hypothenemus hampei* in study sites in Colombia, Ethiopia, Tanzania, and Kenya.

Based on the number of degree-days in the four sites in Colombia, Kenya, Ethiopia and Tanzania (Table S1), the estimated number of generations of *H. hampei* per year ranged from 0.0 to 4.71. The calculated number of *H. hampei* generations for Kilimanjaro (Tanzania) and Chinchiná (Colombia) were very similar, ranging from 2.39 to 4.71, and 2.95 and 4.30, respectively. The number of *H. hampei* generation per year ranged between 2.03–3.13 for Kisii, Kenya (Fig. 4). With 0.0–2.02 the lowest number of beetle generations per year was estimated for Jimma, Ethiopia (Fig. 4).

**Figure 5:**
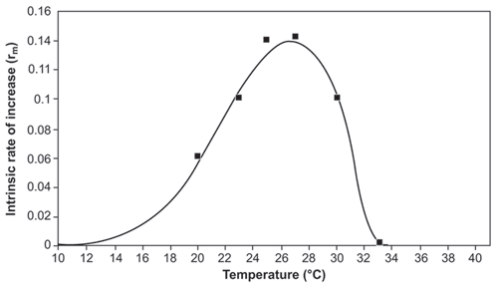
*Hypothenemus hampei* intrinsic rate of increase (r_m_) estimated as function of temperature (°C) using a Gaussian times a Gompertz model.

The Gaussian times a Gompertz model gave a good fit to the r_m_ vs. temperature data sets within the range of 10–33°C (*r* ^2^ = 0.98, *F* = 1592.28 *P*<0.0001; Fig. 5). The fitted parameters of the model were estimated as *r*_max_ = 0.14 (se = 0.003), ρ = 1.17 (se = 0.496), T_opt_ = 26.7 (se = 0.421), Δ = 0.02 (se = 0.004). These analyses suggest that the r_m_ is strongly influenced by temperature.

Calculated data on warming tolerance (WT) and thermal safety margins (TSM) of the insect for the three East African locations showed considerably high variability compared to the Colombian site (Table S2). The two variables were strongly and positively related (*r* = −0.936, *P* = 0.0001). However, the intrinsic rate of increase was significantly negatively correlated with the TSM for the period between 1975 and 2004. For every 1°C rise in T_opt_., *r*_max_ will increase by an average of 8.5%.

## Discussion

Global warming is already affecting the bionomics of arthropods. Detailed studies from the temperate zones on several species report mainly positive effects on insect fitness and distribution range [31], [19], [22], [32]. On the other hand, surprisingly, the potential impact of global warming in tropical insects has only been studied in hematophagous insects such as malaria-transmitting *Anopheles* spp. (Diptera: Culicidae) [33] and the tsetse fly *Glossina pallidipes* Austen (Diptera: Glossinidae) [34]. In this paper, we report the first detailed analysis on the potential effects of climate change on the bionomics of *H. hampei*, the most devastating pest of coffee throughout the world. In a recent paper Deutsch et al. [28] predicted losses in tropical ectotherm biodiversity due to global warming.

In the absence of available long-term abundance data of *H. hampei* from the field in coffee producing areas of Africa and the Americas, we resorted to detailed laboratory studies on the effect of temperatures on the bionomics of the beetle. Previous studies on the basic biology of the insect were either carried out in the field [35]–[37], or in the laboratory with only one temperature regime [e.g.], [ 38]–[40]. Using a recently developed experimental protocol [41] we were able to study the effects of eight different temperature regimes on the bionomics of *H. hampei*. According to Stevens' [42] climatic variability hypothesis, the thermal tolerance of an insect is directly proportional to the climatic variability the organism is exposed to. Our study shows that the prediction of Steven's hypothesis was supported by the case of *H. hampei*. The estimated extremes for *H. hampei* survival are 15 and 32°C. Although our model predicts fastest development of the insect between 27–30°C, there is clear trade-off between development time and reproductive success as previously shown for other insects [43]. The highest rate of survival in colonizing females was recorded at the lowest temperature tested (Table 1).

*H. hampei* attacks and successfully develops on both *C. arabica* and *C. canephora* [44]. Moreover, the coffee berry borer is one of the few herbivores feeding on the endosperm of coffee due to its ability to detoxify caffeine [45], [46]. *Coffea arabica* is believed to have originated in the south-western highlands of Ethiopia, south-east Sudan (Boma Plateau) and the area around Mt. Marsabit in Kenya where it naturally grows as an understory tree in forests [5]. In this area, mean air temperatures range between 18–22°C [13]. In contrast, *C. canephora* is native to a vast area that covers West and West-Central Africa (Cameroon, Congo, Central African Republic, Democratic Republic of Congo, Gabon), North-East and East Africa (Sudan, Tanzania, Uganda), and Southern Africa (Angola). Yet, the exact limit of the centre of origin is difficult to determine because of introduction and naturalization [5]. In this area, mean annual air temperatures are higher than in the Ethiopian highlands [12]. For many years, there has been controversy in the literature about the geographic origin of the pest [47]–[49] and its original host plant(s) [50], [51]. Resolving this mystery might have important implications for future breeding for host plant resistance in coffee, as well as better targeted explorations for natural enemies of the coffee berry borer. Based on our estimates on the thermal tolerance of *H. hampei* it is unlikely that the beetle is endemic to the area around Jimma (Ethiopia) due to the low annual minimum temperatures prevalent there. During an extensive survey, Davidson [51] did not find *H. hampei* in Ethiopia and Damon [29] later speculated that the absence of the pest is due to either specialized natural enemies, resistant varieties of *C. arabica* or exceptionally clean harvest practices in Ethiopian plantations. However, our analysis of 32 years of climatic data from Jimma indicate that before 1984 it was too cold for the insect to complete even one generation per year, but thereafter, because of rising temperatures in the area, the pest is now able to complete 1–2 generations per year/coffee season. This may explain why in a more recent study Mendesil et al. [52] reported widespread occurrence of the coffee berry borer in southwestern Ethiopia.

Campbell et al. [53] emphasized the usefulness of the lower threshold of development and the thermal constant of an insect to elucidate its potential distribution. Similar to *C. arabica*, *C. canephora* is an understory tree of lowland forests. Climatological data from shaded coffee plantations in Central America [54], [55] and East Africa [56] indicate a reduction in temperature between 2–6°C depending on the region, when compared to coffee grown without shade. Considering these findings with our data on thermal tolerance of *H. hampei* and the annual mean temperatures in the area of origin of *C. canephora* [5], we hypothesize that the original host plant of *H. hampei* is likely to be robusta coffee. Since the two coffee species naturally occur in relative proximity in Central and Eastern Africa [5] it could have been possible that the coffee berry borer actively or passively dispersed from its original host plant *C. canephora* to the closely related *C. arabica*. Moreover, in life table studies in the laboratory *H. hampei* performed significantly better on robusta compared to arabica coffee (J. Jaramillo, unpublished data).

How do the findings reported in this paper relate to potential climate change effects on the coffee berry borer? Bale et al. [19] suggest that direct effects of temperature are likely to be stronger and more important than other factors related to climate change such as CO_2_ levels, rainfall pattern, etc. Moreover, data on thermal tolerance of an insect is crucial to predict the possible effects of climate change [e.g.], [57], [21], [22], [58]. In the case of *H. hampei*, average daily temperatures >26°C could lead to a reduction of the maximum intrinsic rate of increase, and, consequently, reduced pest activity in coffee plantations. Over the last three decades, the average daily temperature per year ranged between 17.3–22.3°C for Ethiopia, 18.7–24.5°C for Kenya, 22.3–29.8°C for Tanzania and for Colombia 15.5–29.3°C (data from 1989 to 2007, as *H. hampei* was introduced in 1988 into the country). The potential number of *H. hampei* generations per year was in average 3.4 for Colombia, 3.1 for Kenya, 3.1 for Tanzania and 1.3 for Ethiopia. According to our predictive model, in regions where the actual average daily temperature has not yet reached 26.7°C, every 1°C increase, would also increase the actual *r* towards the maximum value by an average of 8.5%. Since population growth of *H. hampei* is exponentially related to temperature (see Fig. 5), an increase of this magnitude may profoundly influence the pest population dynamics. Thus, areas with higher seasonal temperature close to the optimum development of *H. hampei* would experience high pest pressure as indicated by the higher number of generations of pests during its active period of the year. Because the coffee berry is a finite resource, this could lead to increased borer dispersal, as more females may be competing for oviposition sites. Such a scenario could have devastating effects in coffee growing areas like Colombia where, because of well distributed precipitation leading to multiple flowering of the plants, there is yearlong supply of coffee berries [59]. The problem would be less severe in East Africa where there is a marked and prolonged dry period, with consequent absence of berries in the field for extended periods.

According to Hodkinson [60] the eco-physiology of both insect and plants will predict the future distribution of insect pests when both host plant and herbivore are in close synchrony. Thus, in the case of a highly specialized herbivore like *H. hampei*, the effects of climate change on the insect and the plant cannot be separated. Assad et al. [10] for Brazil, Gay et al. [11] for Mexico and Grid [9] for Uganda predict that even a small increase in temperature due to climate change, will have serious consequences for coffee production in these countries, in some cases rendering production very difficult. Under a climate change scenario, species like *H. hampei*, whose distribution are restricted by both temperature and the availability of their host plants, will follow plant distribution [61]. Coffee is mainly grown in the tropics from 20–25°N to 24°S [61]. Reactions of insects to climate change include, among others, acclimation [62], [63], and changes in latitudinal and altitudinal distribution [64], [65], [66], [26]. A latitudinal expansion in *C. arabica* and *C. canephora* is problematic because both species are highly susceptible to changes in photoperiod, with effects ranging from a marked reduction of their growth phase to an inhibition of flower development [67]. Yet to date coffee is grown in, among others, Nepal (http://www.plantecnepal.com/) and the Yunnan province of China (http://www.yunnancoffee.org/), both areas outside the before mentioned tropical distribution range of *C. arabica* and *C. canephora*. Our data on thermal tolerance of *H. hampei* on the other hand would predict that the pest is well capable to thrive also under sub-tropical conditions. An altitudinal expansion as a coping strategy in a climate change environment is potentially feasible, though there are few areas in the tropics where coffee production could expand in altitude (e.g. the Kilimanjaro area of Tanzania, Mt. Kenya, and the mountain ranges of Colombia), considering that other requirements for successful coffee production like soil type and appropriate rainfall patterns have to be met [6]. For instance, Jaramillo [68] estimated that for the coffee growing area in Colombia an increase in temperature of 1°C would require to move the plantations by 167m in altitude to maintain the same productivity and quality in arabica coffee. Recent data from Uganda and Indonesia show that the coffee berry borer has already expanded its altitudinal distribution range and is now attacking coffee plantations at sites as high as 1,864 m.a.s.l. (Dr. Africano Kangire, National Agricultural Research Organization, Uganda & Dr. Surip Mawardi, Indonesian Coffee and Cocoa Research Institute, pers. comm.)

A proven strategy to alleviate the potentially negative effects of climate, especially warmer temperatures, on coffee production is the introduction of shade trees in coffee plantations [67], [69]. Shade trees mitigate microclimatic extremes and can buffer coffee plants from microclimate variability [70], reduce high solar radiation and buffer detrimental diurnal changes in air temperature and humidity [55], [71], leading to a decrease in the temperature around the coffee berries by up to 4°C [71] under low altitude conditions (i.e., <700 m.a.s.l.), and by up to 2°C under mid to high altitude conditions (i.e., >1,100 m.a.s.l.) [56]. Moreover, Teodoro et al. [72] recently demonstrated that densities of the coffee berry borer were significantly lower in shaded versus un-shaded coffee plantations, possibly because shade coffee agro-ecosystems can serve as a refuge for beneficial arthropods (native and introduced), leading to higher levels of biological control of *H. hampei* [73], [74]. Finally despite lower yields of shaded compared to un-shaded coffee, the berry weight is higher and the quality of coffee produced under shade is better [75], with overall favorable economics for small-scale producers [76].

Our findings indicate that *H. hampei* can survive and reproduce within a broad temperature regime and that the potential number of generations as an indicator of the pest status varies profoundly with daily seasonal temperature. The lower number of generations found in Ethiopia is probably a result of the lower temperature prevalent in the sampling area. Thus we believe the most appropriate way for coffee production systems to cope with climate change is to come back to the origins of coffee as an understory tree in the forests of Africa.

## Materials and Methods

### Insects

Females of the coffee berry borer were obtained from an *H. hampei* stock culture established in July 2005 with beetle-infested coffee berries collected from an organic coffee plantation located in South Kisii (Gucha), Western Kenya (0° 45' 49.85'' S, 34° 43' 1.76'' E). The colony is maintained at the International Center of Insect Physiology and Ecology (*icipe*), Nairobi, Kenya, where the insects are reared on ca. 150 days old coffee berries (*C. arabica* var. Ruiru 11) kept at room temperature (25±1°C), 70% ±5% relative humidity [RH], and a 12∶12 h (L:D) photoperiod. Infested berries were kept inside square plastic containers (40×40×20 cm) with perforated lids (55 mm diameter) covered with insect gauze. The bottom of each container was layered with a 1.5 cm mixture of plaster of Paris and activated charcoal to maintain humidity and prevent the desiccation of the berries and the insects [41].

### Experimental setup and data assessment

The study was conducted at *icipe* laboratories. Organically produced coffee berries (*C. arabica* var. Ruiru 11) ca. 150 days old were collected from the Kiambu district of Central Kenya (1° 10' S, 36° 49' 60 E, 1,723 m.a.s.l.). Once in the laboratory, berries were surface sterilized using the following protocol [77]: the berries were washed with detergent for 15 min, rinsed with tap water, then dipped in a 2% sodium hypochlorite solution for 10 min, rinsed again with sterile distilled water, soaked in a 2% potassium sorbate solution and finally rinsed with sterile distilled water. Subsequently the excess of water was removed with a paper towel and the coffee berries were allowed to dry at room temperature. Afterwards, the berries were placed in round plastic containers (23 cm diameter×6.8 cm depth) (approx. 150 berries per container) and exposed to large numbers of *H. hampei* females from the stock culture. After 2 h of exposure, berries that had been bored by one female were selected and transferred individually into each well of a 12-well microtiter plate (Costar® 3526, Corning Inc., Corning, NY, USA). Each well (23 mm diameter; 20 mm deep) was filled with a 0.5 cm layer of a mixture of plaster of Paris and charcoal [41]. Twelve holes (15 mm dia), coinciding with the wells, were perforated in the lid of every multiwell plate and covered with mesh to allow aeration of the experimental units and to prevent escape of the beetles. The multiwell plates were then transferred to temperature controlled climate chambers (SANYO® MIR-553, Sanyo Electrical Ltd., Japan) set at eight different constant temperatures (15, 20, 23, 25, 27, 30, 33 and 35°C), 80 ±5% RH, and a 12∶12 h (L: D) photoperiod. To keep up the humidity inside the experimental units, distilled sterile water was added to each well every two, three days or daily for multiwell plates kept at 20–30°C, 15°C and 33–35°C, respectively.

Numbers of live and dead *H. hampei* colonizing females as well as their position inside the berries (see below) and number of borer life stages (i.e., eggs, larvae, prepupae, pupae and adults) were assessed daily for periods between 30 and 60 days depending on the temperature.

Four different positions based on the insect location within the berry have been identified by Bustillo et al. [78] as follows: (A), when the female is starting to colonize a new berry but the penetration in the exocarp has not taken place; (B), when the female has penetrated the berry but has not yet reached the endosperm; (C), when the female has started to bore into the endosperm but not to oviposit; and (D), when the female has produced a gallery in the endosperm, and one or more of its immature stages are found inside the gallery.

The evaluations concluded when egg laying by the F2 generation was observed, i.e., between 30 and 60 days after the infestation of the berries depending on the temperature. The coffee berries were dissected under a 10X stereomicroscope and the position of the colonizing female inside the berry was recorded and the numbers of *H. hampei* immature stages were counted. On a daily basis, five berries per temperature and per replicate were destructively sampled and dissected under the stereomicroscope. The experiment was repeated four times over time for insects kept at 15 and 25°C, and three times for insects kept at 20, 23, 27, 30, 33 and 35°C. A total of 5,925 berries/*H. hampei* colonizing females were used, divides as follows: 1,200 for each 15 and 25°C (evaluations for a period of 60 days); 900 for 20°C (evaluations for a period of 60 days); 600 for each 23 and 27°C (evaluations for a period of 40 days); 525 for 30°C (evaluations for a period of 35 days); and 450 for each 33 and 35°C (evaluations for a period of 30 days).

### Climatic data and estimated number of generations of H. hampei at four locations in Africa and South America

Daily climatic data were obtained for four coffee growing areas, with three locations in East Africa (Jimma, Ethiopia; Kisii, Kenya; and Kilimanjaro, Tanzania) and one location in South America (Chinchiná - Colombia) (Fig. SI 1). Precipitation data were used to estimate the yearly blossoming period of the main coffee harvest in the different locations. A single heavy rain (>10 mm rain), followed by a prolonged dry period usually triggers the blossoming of a coffee tree [79]. The physiological development of a coffee berry from flowering to the harvest of the ripe berry takes around 32 weeks or 240 days [80]. *H. hampei* females start to search for suitable coffee berries around 100 days after flowering and oviposit inside the berries usually 20 days later [37]. In the absence of *H. hampei* population dynamics data in all four locations, the findings of [79] and [37] were used to estimate the probable time of *H. hampei* oviposition in the different locations in East Africa and Colombia. Therefore, long-term daily data on temperature (Fig. SI 1) and precipitation (data not shown) in the different locations only for the period between 120–240 days of coffee berry development, together with our laboratory derived data on degree-days for *H. hampei*, were used to estimate the number of potential *H. hampei* generations per year and location.

### Statistical analysis

The mortality/survival and the positions of the colonizing *H. hampei* female inside the coffee berry (A, B, C and D) at each temperature were analyzed using logistic regression (PROC LOGISTIC) [81].

Differences in developmental times, survivorship and life history parameters between temperatures were analyzed by analysis of variance (ANOVA), using the general linear model (PROC GLM) [81]. An *F* test was used to test the significance of mean differences and mean values were separated using the Student Newman Keuls (SNK) test. The significance level was set at *P*≤0.05. Percentages were transformed to arcsine values before analysis. The significance level was set at *P* = 0.05, but back-transformed data are presented in the tables. For estimation of the lower developmental threshold (*T_0_*) which is the intercept over the slope of the regression, i.e., the numbers of day-degrees to complete the pre-reproductive phase and the thermal constant (*Kc*) which is defined as one over the slope, a regression over the linear range of the relationship between temperature (T) and developmental rates [R (T)] of the insect was used [53]. 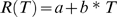
(1)


A modified Logan model [82] by Lactin et al. [83] was used to describe the relationship between temperature and development rate,
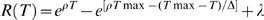
(2)


Where e is the exponential function, T is the temperature in degrees Celsius (°C), ρ, Tmax, Δ and λ are fitted coefficients.

All parameters in nonlinear models were estimated by minimization of the sum of squared residuals. Parameters were tested against 0, based on non-overlap of 95% confidence intervals.

Life table statistics were calculated according to Hulting et al. [84] using SAS, with calculation of confidence intervals for all estimated parameters [85]. The two-sided *t*-tests values, as well as their respective *P* values were computed, and mean values were separated using a pairwise comparison between populations.

For Kilimanjaro (Tanzania), Jimma (Ethiopia), Kisii (Kenya), and Chinchiná (Colombia), the historical degree-days were calculated between 120 and 240 days after flowering. The number of degrees above the threshold degree-days, for a single day are calculated as follows:
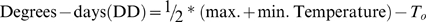



Where Max. and Min. are daily maximum and minimum temperature (°C). If Min. temperature was lower than the minimum threshold *T_0_* (see Table 2), then Min. temperature was set to minimum threshold. If Max. temperature was higher than the maximum threshold 33°C (see Table 2), then Max. temperature was set to 33°C. The estimated number of *H. hampei* generations per year was calculated by dividing historical cumulative degree-days per year and location by the experimental estimation of *Kc*.

### Warming Tolerance and Thermal Safety Margins of *H. hampei*

Warming tolerance (average amount of environmental warming an ectotherm can tolerate before performance drops to fatal levels) and thermal safety margins (temperature at which the performance of the organism will start to decrease) were calculated according to Deutsch et al. [28] as follows:
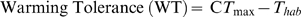



and
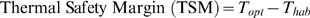



Where CT_max_ is the critical thermal maximum of *H. hampei*, T_opt_ is *H. hampei* thermal optimum and T_hab_ is the current climatological temperature of the organism's habitat.

The intrinsic rate of increase Rm(T) was fitted to the climatic data of the four locations using a Gaussian times a Gompertz function to predict the physiological consequences of climate change on the fitness of *H. hampei*:
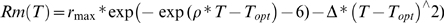



Where r_max_ and T_opt_ are the predictions where the insect has a maximal fitness, ρ represents the increasing part of the population growth rate curve and Δ the declining part of the curve.

## Supporting Information

Table S1 Number of degree days available to the coffee berry borer (above 14.9C) in three locations in East Africa and one in South America.
(0.08 MB DOC)

Table S2 Warming Tolerance (WT) and Thermal Safety Margin (TSM) (calculated after Deutsch et al., 2008) for *Hypothenemus hampei* in three locations in East Africa and one in South America.
(0.12 MB DOC)

Abstract S1 Spanish Abstract - Resumen
(0.03 MB DOC)

Abstract S2 French abstract - resume
(0.03 MB DOC)
