# Supplementary material for: Thermal Tolerance of the Coffee Berry Borer Hypothenemus hampei: Predictions of Climate Change Impact on a Tropical Insect Pest
Source: PLoS One. 2009 Aug 3;4(8):e6487. doi: 10.1371/journal.pone.0006487 (PMC2715104; doi:10.1371/journal.pone.0006487)
Supplement: Table S1 — Number of degree days available to the coffee berry borer (above 14.9C) in three locations in East Africa and one in South America. (0.08 MB DOC) [file pone.0006487.s001.doc]

SI 1. Number of degree days available to the coffee berry borer (above 14.9°C) in four locations in East Africa and one in South America.

| Year | Location | | | |
| --- | --- | --- | --- | --- |
|  | Chinchiná, Colombia | Kilimanjaro, Tanzania | Jimma, Ethiopia | Kisii, Kenya |
| 1974 | -* | 688.42 | -** | -** |
| 1975 | -* | 838.51 | -** | -** |
| 1976 | -* | 875.73 | -** | -** |
| 1977 | -* | 927.20 | 235.50 | -** |
| 1978 | -* | 821.45 | 105.40 | -** |
| 1979 | -* | 667.73 | -** | -** |
| 1980 | -* | -** | 178.00 | -** |
| 1981 | -* | 851.40 | 218.90 | -** |
| 1982 | -* | 688.10 | 186.80 | -** |
| 1983 | -* | 824.64 | 248.49 | -** |
| 1984 | -* | 832.60 | 352.72 | -** |
| 1985 | -* | 773.71 | 293.66 | 587.44 |
| 1986 | -* | 709.32 | 374.67 | 582.50 |
| 1987 | -* | 995.70 | 503.20 | 633.68 |
| 1988 | -* | 823.71 | 338.55 | 637.97 |
| 1989 | 775.01 | 846.90 | 402.07 | 673.85 |
| 1990 | 906.35 | 676.61 | 303.05 | 533.85 |
| 1991 | 1128.68 | 746.59 | 351.34 | 554.74 |
| 1992 | 888.85 | 712.28 | 274.72 | 593.23 |
| 1993 | 841.40 | -** | -** | 726.17 |
| 1994 | 848.81 | -** | 460.49 | 772.98 |
| 1995 | 949.74 | -** | -** | 823.15 |
| 1996 | 1002.68 | -** | -** | 774.59 |
| 1997 | 946.24 | 628.37 | -** | 828.34 |
| 1998 | 896.65 | 706.69 | 393.69 | 647.23 |
| 1999 | 594.88 | -** | -** | -** |
| 2000 | 833.90 | 799.04 | -** | -** |
| 2001 | 877.90 | 825.56 | -** | -** |
| 2002 | 921.54 | 1082.46 | -** | 636.19 |
| 2003 | 885.65 | 799.80 | -** | 769.26 |
| 2004 | 861.50 | 862.86 | -** | 672.84 |
| 2005 | 886.80 | -** | -** | -** |
| 2006 | 941.64 | 1237.57 | 532.72 | -** |
| 2007 | 876.20 | 982.02 | 465.96 | 639.25 |

* *H. hampei* not yet present in this coffee growing area of Colombia

** Climatic data not available. Either the blossoming period of the coffee plants or the number of degrees days could not be estimated for these years.
