# Supplementary material for: Thermal Tolerance of the Coffee Berry Borer Hypothenemus hampei: Predictions of Climate Change Impact on a Tropical Insect Pest
Source: PLoS One. 2009 Aug 3;4(8):e6487. doi: 10.1371/journal.pone.0006487 (PMC2715104; doi:10.1371/journal.pone.0006487)
Supplement: Table S2 — Warming Tolerance (WT) and Thermal Safety Margin (TSM) (calculated after Deutsch et al., 2008) for Hypothenemus hampei in three locations in East Africa and one in South America. (0.12 MB DOC) [file pone.0006487.s002.doc]

SI 2. Warming Tolerance (WT) and Thermal Safety Margin (TSM) (calculated after Deutsch *et al*., 2008) for *Hypothenemus hampei* in three locations in East Africa and one in South America.

|  | **Location** | | | | | | | |
| --- | --- | --- | --- | --- | --- | --- | --- | --- |
| Year | Chinchiná, Colombia | | Kilimanjaro, Tanzania | | Kisii, Kenya | | Jimma, Ethiopia | |
|  | WT | TSM | WT | TSM | WT | TSM | WT | TSM |
| 1974 | -* | -* | 4.50 | 1.50 | -** | -** | -** | -** |
| 1975 | -* | -* | 4.60 | 1.60 | -** | -** | -** | -** |
| 1976 | -* | -* | 6.27 | 3.27 | -** | -** | -** | -** |
| 1977 | -* | -* | 5.16 | 2.16 | -** | -** | 10.58 | 7.58 |
| 1978 | -* | -* | 3.77 | 0.77 | -** | -** | 10.61 | 7.61 |
| 1979 | -* | -* | 5.61 | 2.61 | -** | -** | -** | -** |
| 1980 | -* | -* | 7.22 | 4.22 | -** | -** | 11.68 | 8.68 |
| 1981 | -* | -* | 5.77 | 2.77 | -** | -** | 11.58 | 8.58 |
| 1982 | -* | -* | 6.55 | 3.55 | -** | -** | 11.39 | 8.39 |
| 1983 | -* | -* | 7.66 | 4.66 | -** | -** | 11.61 | 8.61 |
| 1984 | -* | -* | 5.66 | 2.66 | -** | -** | 11.94 | 8.94 |
| 1985 | -* | -* | 5.33 | 2.33 | 7.27 | 4.27 | 11.63 | 8.63 |
| 1986 | -* | -* | 6.22 | 3.22 | 5.49 | 2.49 | 11.86 | 8.86 |
| 1987 | -* | -* | 3.11 | 0.11 | 8.49 | 5.49 | 11.37 | 8.37 |
| 1988 | -* | -* | 5.38 | 2.38 | 10.72 | 7.72 | 11.58 | 8.58 |
| 1989 | 9.21 | 6.21 | 7.55 | 4.55 | 11.33 | 8.33 | 11.94 | 8.94 |
| 1990 | 8.43 | 5.43 | 4.16 | 1.16 | 8.60 | 5.60 | 11.74 | 8.74 |
| 1991 | 8.33 | 5.33 | 5.83 | 2.83 | 11.33 | 8.33 | 11.37 | 8.37 |
| 1992 | 8.48 | 5.48 | 5.38 | 2.38 | 8.55 | 5.55 | 11.01 | 8.01 |
| 1993 | 8.74 | 5.74 | 3.50 | 0.50 | 9.49 | 6.49 | -** | -** |
| 1994 | 8.74 | 5.74 | 3.10 | 0.10 | 7.72 | 4.72 | 11.01 | 8.01 |
| 1995 | 9.06 | 6.06 | 5.38 | 2.38 | 9.22 | 6.22 | -** | -** |
| 1996 | 9.20 | 6.20 | 3.60 | 0.60 | 7.44 | 4.44 | -** | -** |
| 1997 | 8.39 | 5.39 | 4.61 | 1.61 | 9.88 | 6.88 | -** | -** |
| 1998 | 8.30 | 5.30 | 0.22 | -2.78 | 9.66 | 6.66 | 10.19 | 7.19 |
| 1999 | 9.40 | 6.40 | 5.11 | 2.11 | -** | -** | -** | -** |
| 2000 | 9.27 | 6.27 | 5.27 | 2.27 | -** | -** | -** | -** |
| 2001 | 8.81 | 5.81 | 2.22 | -0.78 | 8.60 | 5.60 | -** | -** |
| 2002 | 8.41 | 5.41 | 6.66 | 3.66 | 7.44 | 4.44 | -** | -** |
| 2003 | 8.60 | 5.60 | 3.11 | 0.11 | 8.33 | 5.33 | -** | -** |
| 2004 | 8.70 | 5.70 | 5.72 | 2.72 | 10.94 | 7.94 | -** | -** |
| 2005 | 8.61 | 5.61 | -** | -** | -** | -** | -** | -** |
| 2006 | 8.61 | 5.61 | 6.98 | 3.98 | -** | -** | 9.36 | 6.36 |
| 2007 | 8.83 | 5.83 | 4.7 | 1.7 | 7.9 | 4.39 | 9.27 | 6.27 |

* *H. hampei* not yet present in this coffee growing area of Colombia

** Climatic data not available. Either the blossoming period of the coffee plants or the number of degrees days could not be estimated for these years.
