# Supplementary material for: Thermal Tolerance of the Coffee Berry Borer Hypothenemus hampei: Predictions of Climate Change Impact on a Tropical Insect Pest
Source: PLoS One. 2009 Aug 3;4(8):e6487. doi: 10.1371/journal.pone.0006487 (PMC2715104; doi:10.1371/journal.pone.0006487)
Supplement: Abstract S1 — Spanish Abstract - Resumen (0.03 MB DOC) [file pone.0006487.s003.doc]

Supporting material 3. Spanish abstract

**RESUMEN**

Se predice que el cambio climático global y la variabilidad climática natural afectarán seriamente el cultivo del café (*Coffea arabica* y *C. canephora*) en el mundo. En este estudio se determinó la tolerancia térmica de la broca del café, *Hypothenemus hampei*, considerado como el insecto plaga más dañino del café, y se estimaron los posibles efectos del calentamiento climático en las poblaciones del insecto utilizando información de temperatura de localidades situadas en áreas cafeteras de Colombia, Kenia, Tanzania, y Etiopia. Para esto, inicialmente se evaluó el efecto de ocho regímenes de temperatura (15, 20, 23, 25, 27, 30, 33 y 35 ºC) sobre los parámetros poblacionales de *H. hampei*. El desarrollo óptimo de huevo a adulto ocurrió entre 20-30 ºC. Usando regresiones lineales y un modelo Logan modificado, se estimaron las temperaturas base inferior y superior para el desarrollo, encontrándose valores de 14,9 °C y 32,0 °C, respectivamente. En Kenia y Colombia, el número de generaciones anuales de la broca del cafeto se incrementaron y se correlacionaron positivamente con la tolerancia calórica (Warming Tolerance). Al analizar 32 años de información climática de Jimma (Etiopía) se encontró que antes de 1984 las condiciones de temperatura eran demasiado frías para que *H. hampei* pudiera completar su desarrollo, incluso una generación por año, pero posteriormente, debido al aumento de la temperatura en esa área, se desarrollaron de 1 a 2 generaciones/año-período de fructificación del café. Los cálculos de tolerancia calórica y del límite de seguridad termal (Thermal Safety Margin) de *H. hampei* para los tres países africanos mostraron una alta variabilidad al compararlos con la localidad en Colombia. Nuestro modelo indica que por cada 1 ºC de aumento en el óptimo térmico (T_opt._), la tasa máxima de incremento intrínseco (*r*_max_) se incrementa en promedio un 8.5 %. Se discuten los efectos del cambio climático sobre la futura distribución mundial de la broca del café y las posibles estrategias de adaptación a este cambio.

***Palabras claves:*** *Hypothenemus hampei*, tasa máxima de incremento intrínseco, tolerancia térmica, broca del café, *Coffea arabica*, *Coffea canephora*, temperatura, cambio climático, trópico.
