# Supplementary material for: Thermal Tolerance of the Coffee Berry Borer Hypothenemus hampei: Predictions of Climate Change Impact on a Tropical Insect Pest
Source: PLoS One. 2009 Aug 3;4(8):e6487. doi: 10.1371/journal.pone.0006487 (PMC2715104; doi:10.1371/journal.pone.0006487)
Supplement: Abstract S2 — French abstract - resume (0.03 MB DOC) [file pone.0006487.s004.doc]

Supporting Material 4. French abstract

**RESUME**

Les prévisions indiquent que la culture du caféier va être sévèrement affectée par les changements climatiques. Nous avons déterminé la tolérance thermique du scolyte du caféier, *Hypothenemus hampei,* principal ravageur à l’échelle mondiale, et avons estimé les effets possibles du changement climatique à l’aide de données de Colombie, du Kenya, de Tanzanie et d’Ethiopie. Pour cela nous avons étudié les effets de 8 régimes de température (15, 20, 23, 25, 27, 30, 33 et 35ºC) sur les principaux paramètres de développement de *H. hampei*. Le développement est normal entre 20-30ºC. En utilisant une régression linéaire et le modèle de Logan modifié, les seuils de développement minima et maxima ont été respectivement estimés à 14,9 et 32°C. Au Kenya et en Ethiopie, le nombre de générations annuelles du ravageur est significativement corrélé à la tolérance thermique. L’analyse de 32 années de données climatiques de Jimma (Ethiopie) indique qu’avant 1984, la température était trop froide pour permettre le développement d’une seule génération d’*H hampei* mais que, par la suite, en raison de l’augmentation des températures, il pouvait atteindre 1 à 2 générations par an. Le calcul de la tolérance thermique et des marges de sécurité thermique d’*H hampei* dans les trois pays d’Afrique de l’Est montre une importante variabilité, comparé à la Colombie. Le modèle indique que, pour toute augmentation d’1°C de l’optimum thermique (T_opt._), le taux intrinsèque d’accroissement maximum (*r*_max_) augmentera en moyenne de 8,5%. Nous discutons des effets des changements climatiques sur la future zone de distribution d’*H. hampei* et sur ses éventuelles stratégies d’adaptation.

***Mots clés:*** *Hypothenemus hampei*, parameters de développement, tolérance thermique, scolyte du café, *Coffea arabica*, *Coffea canephora*, température, changement climatique, tropiques.
